# From lobotomy to connectomics: a critical review of contemporary psychosurgery

**DOI:** 10.3389/fpsyt.2026.1785333

**Published:** 2026-04-23

**Authors:** Victor González, Jordi Rumià, Marta Brell, Carolina Roset, José de Miguel, Susana Arboleya, Iratxe Aguirre, Daniel Alegre, Victor Goliney, Javier Ibáñez

**Affiliations:** 1 Department of Neurosurgery, Hospital Son Espases, Palma, Spain; 2 Department of Neurosurgery, Hospital Clínic de Barcelona, Barcelona, Spain; 3 Department of Psychiatry, Hospital Son Espases, Palma, Spain

**Keywords:** connectomics, deep brain stimulation, neuromodulation, obsessive-compulsive disorder, psychosurgery, treatment-resistant depression

## Abstract

Contemporary psychosurgery is increasingly regarded less as an invasive surgical discipline and more as a circuit-based medicine. This narrative critical review traces the evolution from the extensive frontal lesions of the early twentieth century to contemporary techniques, in which focal ablation and reversible neuromodulation form part of a shared therapeutic armamentarium. Building on the classical history—from trephinations and lobotomies to stereotaxy—we outline a functional neuroanatomical framework that integrates thelimbic system, basal ganglia, prefrontal cortex, and monoaminergic systems, conceptualized as networks in which emotional and cognitive symptoms are generated and maintained. We then introduce the foundations of connectomics (diffusion tensor imaging, functional MRI, and graph-theoretical models) and their contributions to redefining targets not as isolated points but as nodes within fronto-limbic networks implicated in psychopathology. On this substrate, we review the main contemporary techniques: ablative approaches (radiofrequency, radiosurgery, laser ablation, focused ultrasound) and stimulation-based interventions (deep brain stimulation, vagus nerve stimulation, cortical stimulation, and transcranial magnetic stimulation), emphasizing their mechanisms, advantages, and limitations. The core of the article is an indication and circuit-based synthesis of evidence in obsessive- compulsive disorder, treatment-resistant major depression, and other selected conditions (eating disorders, addictions, Tourette syndrome, aggression, schizophrenia, and post-traumatic stress disorder), with particular attention to candidate profiles, clinical outcomes, and integration with psychotherapeutic and pharmacological strategies. We subsequently explore how these developments may converge into a model of precision psychiatry built around symptom dimensions, connectomic-guided targeting, biomarker-informed decision-making, and adaptive stimulation, while also considering the major ethical and organizational issues they raise. Overall, we propose understanding contemporary psychosurgery as a network-based, gradual, and multimodal intervention intended for carefully selected subgroups of treatment- refractory patients whose core symptom clusters might be linked to potentially modifiable white-matter hubs.

## 1 Introduction

Severe and persistent psychiatric disorders remain one of the leading causes of disability, suffering, and healthcare resource utilization. Despite the expansion of pharmacological and psychotherapeutic armamentaria, a proportion of patients continue to present refractory symptoms, marked functional impairment, and a high risk of chronicity or suicide. In this context, interest in psychosurgery re-emerges cyclically: a discipline burdened by historical stigma and associated in the collective imagination with the mass lobotomies of the mid- twentieth century, yet profoundly transformed in its goals, methods, and scientific foundations.

The development of stereotactic neurosurgery, deep brain stimulation, and modern ablative and neuroimaging techniques has enabled a shift from broad, poorly selective lesions to interventions targeting nodes and pathways within fronto–limbic circuits. Psychosurgery is no longer considered an isolated last resort, but rather part of a multimodal approach that includes rigorous candidate selection, interdisciplinary discussion, connectivity-based planning, and long- term follow-up with device programming, psychotherapy, and psychosocial rehabilitation.

This article is conceived as a targeted narrative critical review rather than a formal systematic review, focusing on neurosurgical interventions for severe and treatment-resistant psychiatric disorders in which ablative procedures or deep brain stimulation (DBS) have been used in a clinically meaningful way. The literature discussed was identified through non-systematic searches of major databases (primarily PubMed), with emphasis on landmark clinical studies, recent systematic reviews, and work explicitly linking clinical outcomes to brain networks and connectomic models. Given the heterogeneity of study designs, we prioritized landmark trials, prospective cohorts, and high-quality observational series, complemented by recent systematic reviews when available. Across indications, we summarize the strength of evidence using a pragmatic hierarchy based on study design (randomized controlled trials, RCTs; prospective studies, retrospective cohorts, and case series).

Throughout this review, we use contemporary psychosurgery in a broad sense to refer to psychiatric neuromodulation and stereotactic interventions targeting dysfunctional circuits. While our main focus is on invasive stereotactic procedures, we briefly discuss non-invasive neuromodulation (e.g., rTMS) as part of the therapeutic continuum that typically precedes invasive approaches in treatment-refractory patients.

Our central thesis has two main components. First, we maintain that psychiatric neuromodulation should be guided primarily by symptoms rather than by categorical DSM diagnoses, which often aggregate heterogeneous phenomena. Second, we suggest that optimal surgical targets are best conceptualized as white-matter convergence zones or connectomic hubs, instead of grey-matter nuclei, because these hubs represent critical points where relevant circuits converge and can be effectively modulated.

Accordingly, this article has three main aims. First, to review the historical evolution from non- selective ablation to current techniques, highlighting the milestones that account for both its excesses and its subsequent decline and partial resurgence. Second, to provide a framework of functional and circuit-based neuroanatomy that helps explain why certain targets are plausible for intervening on specific symptoms. Third, to synthesize the available indication-specific evidence and to discuss the ethical, regulatory, and organizational challenges of a contemporary psychosurgery guided by connectomics and by the pursuit of precision psychiatry.

The article is organized as follows. We first provide a brief historical overview of psychosurgery. We then summarize the main functional neuroanatomy and large-scale circuits relevant to psychiatric symptoms, followed by a concise description of current ablative and neuromodulatory techniques. The core of the review is an evidence-based synthesis of indications and circuits for major symptom clusters and disorders, followed by a discussion of ethical, regulatory, and organizational issues and future directions for connectomics-guided precision psychiatry.

## 2 Brief historical overview

Psychosurgery predates the term itself. Archaeological and textual evidence indicate that trephination was performed in prehistory and classical antiquity, sometimes for magico-religious purposes, and probably constitutes the oldest surgical intervention (1). In the Middle Ages, Roger of Parma, in his Practica Chirurgiae (1170), proposed for the first time a surgical indication in the psychiatric domain, such as mania or melancholia. The early modern period added new nuances: treatises such as Robert Burton’s The Anatomy of Melancholy (1620) still referred to trephination for neuropsychiatric disorders (2), while the study of the “modern brain” began to take shape through advances in anatomy, histology, and bioelectricity. In the nineteenth century, new ideas regarding the localization of function were fueled by the case of Phineas Gage (3) and by the work of figures such as Paul Broca and Carl Wernicke, who linked clinical phenomena to post-mortem findings in patients with trauma or epilepsy. In parallel, the birth of modern neurosurgery, led by Viktor Horsley and Harvey Cushing, made the first intracerebral procedures technically feasible and progressively safer.

At the beginning of the twentieth century, however, psychiatry faced a vast therapeutic gap. Scarce resources and overcrowded asylums meant that patients lived in poor conditions. At the same time, alienists experimented with several therapies, including insulin coma, pharmacologically induced convulsive shocks, malariotherapy, and early forms of electroconvulsive therapy (ECT) (4–6), all introduced in a context of limited therapeutic options. Among these, ECT has evolved substantially over time and remains widely used today, with well-established efficacy in several psychiatric disorders (7).

Within this context, Gottlieb Burckhardt attempted cortical resections in psychotic patients, with limited success (8), while clinicians such as Wilder Penfield and John Fulton reported observations on cortical physiology that reinforced the association between psychic and behavioral aspects with the frontal lobes (9, 10). The decisive step came with the Portuguese neurologist Egas Moniz, who, together with neurosurgeon Almeida Lima, introduced frontal leucotomy with the aim of “unblocking” fixed ideas and overwhelming affects in patients with various severe psychiatric disorders (11). The procedure consisted of lesioning the prefrontal white matter tracts through small frontal burr holes, partially disconnecting the frontal cortex from its thalamic and limbic connections. Initial variants used alcohol injection; later versions produced a mechanical lesion with a leucotome. Outcomes were heterogeneous: in some patients, anxiety and agitation decreased, whereas in others apathy, abulia, personality changes, or cognitive deterioration emerged (12).

Moniz’s work was initially met with skepticism in Europe, but shortly thereafter, Walter Freeman and James Watts popularized the procedure in the United States, redefining access points and lesion strategies. By 1942, they had already performed 200 lobotomies, the year in which their monograph *Psychosurgery* was published (13). Given the overcrowding of psychiatric institutions at the time (14), Freeman promoted more expedient versions, including the transorbital.

lobotomy, and advocated wide adoption of the procedure with lax indications and interventions performed outside the operating room, sometimes without a neurosurgeon (1). Despite initial recognition by the media and public opinion, this high-volume (15)- up to 60,000 procedures- and low-supervision phase explains the so-called “excesses of Freeman,” which fueled the subsequent ethical and regulatory backlash that continues to stigmatize these procedures (16, 17).

In response, an increasing number of American and European neurosurgeons sought to refine the focus by proposing more selective approaches. Lesions of white matter bundles were described and performed in a more moderate fashion (cortical undercutting) (18), as well as limited frontal resections (topectomies) (19). With Spiegel and Wycis, the stereotactic frame was introduced in humans, allowing lesions in deep structures according to an external coordinate system. They proposed lesioning the mediodorsal thalamic nucleus as part of the presumed aberrant circuit in psychiatric disorders (20). Moreover, prior animal experimentation helped redefine structures relevant to the surgical treatment of psychiatric disorders—such as the anterior limb of the internal capsule (ALIC) and the cingulate gyrus (21) —targets that remain in use today.

However, from the 1950s onwards, the arrival of psychotropic drugs, such as chlorpromazine and haloperidol (22), together with growing awareness of lobotomy excesses - amplified by literary and film works such as One Flew Over the Cuckoo’s Nest and Suddenly, Last Summer – and the hopes placed in pharmacotherapy, led to a steep decline in psychosurgery and to increasing criticism and pessimism in both lay press and scientific journals (23). Moreover, during the same period, ECT also evolved with the introduction of general anesthesia, muscle relaxants, and improved cardiac monitoring, which greatly enhanced the safety and tolerability of the procedure, consolidating its role as an effective treatment for severe mood disorders.

The 1970s saw a very limited resurgence of surgical approaches, motivated by therapeutic ceilings and adverse effects of psychotropic medications. This period was characterized by improvements in stereotactic techniques, driven by Spiegel and Wycis in the United States and Talairach in France, which enabled more precise and spatially restricted lesions; capsulotomy and cingulotomy became the most frequently employed procedures (24). In addition, the Swedish neurosurgeon Lars Leksell promoted the use of gamma radiation to lesion these structures, obviating the need for intracranial electrodes (25, 26). The main psychiatric indications were severe anxiety and depression, as well as severe obsessive neuroses, and outcome assessment improved with the incorporation of neuropsychological testing. During this decade, clinical trials involving recording and stimulation of various limbic structures across a wide range of psychiatric conditions were also initiated, building on experimental work that had accompanied stereotaxy since its inception (27). These developments reawakened controversies in medical ethics (28) and gave rise to theories about “psychocontrolled” individuals and societies (29).

**Figure 1:**
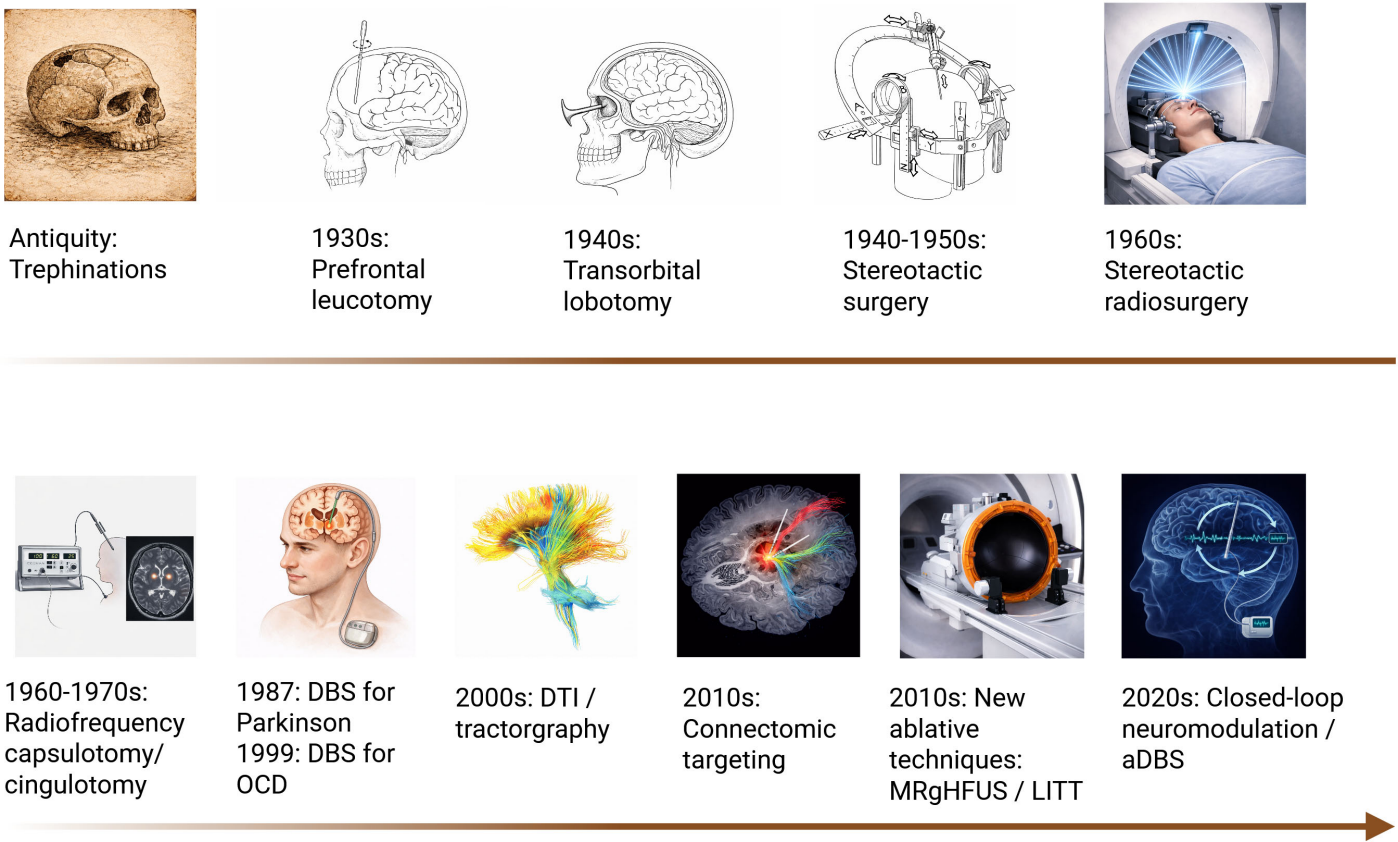
Historical milestones in the development of psychosurgery. Timeline depicting the evolution from ancient trephination to contemporary neuromodulation. DBS, deep brain stimulation; DTI, diffusion tensor imaging; MRgFUS, MR-guided focused ultrasound; LITT, laser interstitial thermal therapy; aDBS, adaptive deep brain stimulation.

By the late 1980s, interest in psychosurgery had waned markedly. Only a few highly specialized centers continued to treat patients. However, a milestone in 1987 would change the course of the field: for the first time, a parkinsonian tremor was successfully treated by DBS of the thalamus (30). This success was subsequently replicated in other movement disorders, such as essential tremor and dystonia (31, 32). High-frequency stimulation delivered by intracranial electrodes appeared to produce lesion-like effects in a reversible and adjustablemanner, theoretically offering advantages over the ablative procedures. A decade later, DBS neuromodulation was extrapolated from movement disorders to psychiatry. In 1999, the Belgian group led by Nuttin reported stimulation of the ALIC in a patient with obsessive– compulsive disorder (OCD) refractory to medical treatment (33), marking the beginning of contemporary psychosurgery. The historical evolution of psychosurgical interventions, from ancient trephination to modern strategies, is summarized in **Figure 1**.

## 3 Functional neuroanatomy of emotion and cognition

### 3.1 Main components of the limbic circuitry

When one asks where emotions “reside,” the answer does not point to a single structure but to a set of circuits that process different types of information and contain key nodes that integrate them. The limbic system and its connections constitute the structural backbone involved in stimulus detection, integration, response generation, and emotional control.

The term limbic system was first coined by Paul Broca, who described a set of structures along the medial border (limbus) of the hemispheres, including the cingulate gyrus and the hippocampus (34). Broca attributed instinctive behavior to the limbic lobe, in opposition to the more “evolved” cognitive functions of the cortex. Decades later, Papez and MacLean added the amygdala, the fornix columns, and the hypothalamus (35, 36), redefining the limbic circuit as a substrate for emotional processing, although it is now understood to be related to mnemonic processes. Additional regions—prefrontal cortex, insula, thalamus, and basal ganglia—were progressively incorporated into the “emotional machinery” supporting the notion of interconnected circuits and networks (37). Today, emotions are understood to arise from dynamic interactions among structures that integrate sensory, cognitive, autonomic, and endocrine information to generate adaptive responses of varying complexity (38, 39).

#### 3.1.1 Prefrontal cortex

The prefrontal cortex (PFC) represents the highest cortical center for the conscious integration and regulation of emotion. For anatomical and didactic purposes, the PFC is usually divided into the dorsolateral prefrontal cortex (DLPFC), the orbitofrontal cortex (OFC), and the anterior cingulate cortex (ACC), with distinct but overlapping functions. The DLPFC supports executive functions and the cognitive control of emotion, enabling the reappraisal of stimuli and the inhibition of impulsive responses (40). Hypoactivity in this region has been consistently associated with major depression and anxiety disorders (41, 42) and has become a key therapeutic target for repetitive transcranial magnetic stimulation (rTMS) (43).

The OFC integrates emotional and motivational information; its more medial portion appears to be more engaged in reward-driven behaviors (44), whereas the lateral portion is more involved in cognitive and evaluative processes (45). Orbitofrontal hyperactivity is a typical finding in OCD, and effective treatment, regardless of modality, tends to normalize this pattern on functional imaging (46–48). The ACC shares functions with the OFC in emotional, motivational, and cognitive tasks, such as error detection, anticipation, and conflict monitoring (49). Its subgenual portion (subcallosal cingulate) is crucial in treatment-resistant depression (TRD), where hyperactivity has been demonstrated in PET and fMRI studies (50), and has become a classic target for DBS in this indication.

#### 3.1.2 Temporal lobe: amygdala and hippocampus

The amygdala can be described as an epicenter for rapid, largely unconscious emotional processing (51). It receives inputs directly from the thalamus and from associative sensory cortices, supporting both rapid automatic survival responses and more complex emotional interpretations (52). After processing, it projects via the stria terminalis and its nucleus, the bed nucleus of the stria terminalis (BNST), to the hypothalamus and brainstem, giving rise to the autonomic and endocrine manifestations of certain emotions. It is thus a convergence structure where sensory information is endowed with affective meaning and translated into biological and behavioral responses.

Amygdalar nuclei are essential for threat detection and conditioned fear (53, 54), but also participate in processing rewarding and social stimuli (55). Electrical stimulation of the amygdala elicits intense autonomic responses (56), whereas bilateral destruction results in the Klüver-Bucy syndrome, characterized by hypersexuality, apathy, and loss of fear (57). In clinical practice, there has been renewed interest in unilateral amygdalar lesioning in extreme cases of refractory aggression (58, 59).

The hippocampus, closely linked to the amygdala, acts as a contextual modulator of emotion and is crucial for declarative memory. It contributes to determining whether an experience is consolidated as an emotional memory (60) and to assigning affective meaning to memories (61, 62). Prolonged exposure to elevated cortisol due to chronic stress can lead to hippocampal atrophy, a phenomenon well documented in depression and post-traumatic stress disorder (PTSD) (63, 64).

#### 3.1.3 Hypothalamic–pituitary axis

The hypothalamic–pituitary axis constitutes the autonomic pathway of emotion, translating affective states into bodily responses. It receives signals from the limbic system and activates both the autonomic nervous system and the adrenal cortex (65). Through these pathways, it produces visceral changes (heart rate, blood pressure, metabolic activity) that accompany emotional states, while activation of the CRH–ACTH axis induces cortisol synthesis and the classic stress response (66). When this activation becomes prolonged, hippocampal synaptic plasticity can be altered, and vulnerability to depressive disorders increased (67).

#### 3.1.4 Insular cortex

The insular cortex is a key bridge between physiological and emotional processes. The anterior insula transforms physiological signals into conscious perceptions (68), while the posterior insula is mainly associated with somatosensory and viscerosensory processing (69). In this way, the insula adds an interoceptive dimension to emotion, enabling recognition of visceral sensations and linking them to affective states. It has also been implicated in addiction mechanisms (70, 71) and in risk-based decision-making (72, 73), underscoring its role in integrating bodily signals, subjective experience, and adaptive behavior. In a simplified circuit, the amygdala converts sensory information into autonomic and endocrine manifestations via the hypothalamic– pituitary axis, and these physiological changes are detected by the insula, bringing them into conscious awareness.

#### 3.1.5 Thalamus and basal ganglia

The basal ganglia, traditionally linked to motor control, also fulfill emotional and motivational functions. They are a key component of the cortico–striato–thalamo–cortical (CSTC) loops,

organized into partially segregated motor, associative, and limbic circuits (74). The striatum (caudate/putamen) is the main gateway for cortical information: the dorsal striatum processes motor and cognitive information, whereas the ventral striatum regulates emotions and reward- driven behaviors (75). At the ventral junction between caudate and putamen lies the *nucleus accumbens* (NAc), a specialized part of the basal ganglia histologically divided into *core* (motor, extrapyramidal) and *shell* (limbic). The NAc is the principal node of the dopaminergic reward circuit, where afferents from the ventral tegmental area (VTA) located in the mesencephalon,

and various limbic structures converge. It has long been conceptualized as an interface between motivation and action (76), participating in motivational behaviors and in the selection and adaptation of responses (77, 78). Neuromodulation of the NAc continues to be investigated in OCD, TRD, and addictions (79, 80). A similar dorsal–ventral parcellation exists in the globus pallidus (GPi/GPe) and subthalamic nucleus (STN), each with motor, associative, and limbic territories (81).

The thalamus is the main relay structure of the central nervous system, integrating motor, somatic, and sensory information (except olfaction) through reciprocal cortical connections. The anterior, ventral anterior, and mediodorsal nuclei are particularly relevant for memory and for cognitive and emotional processing. Thalamic–prefrontal tracts that run through the ALIC are essential for communication between the prefrontal areas and limbic regions. Alterations in these pathways have been implicated in depression and OCD and provide the anatomical basis for anterior capsulotomy and ALIC-DBS.

### 3.2 Emotion circuits

For much of the twentieth century, researchers sought “the place” where emotions resided. It is now clear that there is no single emotional center, but rather interconnected circuits with key nodes integrating memory, motivation, cognition, and autonomic regulation in relation to emotional processes.

In 1937, James Papez described a circuit that he proposed as the primary anatomical substrate of emotion (35). Although now considered partial, it laid the groundwork for modern mnemonic circuit models. In the Papez circuit, information flows diffusely from the cortex to the entorhinal cortex (gateway to the hippocampus), then through the fornix columns to the mammillary bodies of the hypothalamus, ascends via the mammillothalamic tract (MMT) to the anterior thalamic nucleus, and returns to the cingulate cortex, which projects back to the entorhinal cortex. This “anatomical carousel” allows information to loop back to its cortical origin through iterative repetitions, thereby contributing to memory encoding. Animal experimentation and clinical practice have shown that bilateral hippocampal lesions or resections produce anterograde amnesia (82).

The connection between memory and emotion can be explained by the circuit’s links to the hypothalamus and the amygdala, which were almost absent from the original model. Fear- conditioning experiments in animals and humans demonstrate that the amygdala acts as an “alarm center” linking sensory signals to defensive responses, often before full awareness (83). Stress accompanying certain life events triggers catecholamine release by the amygdala, which acts on the hippocampus and tags some memories as particularly salient (84). However, when stress is intense and prolonged, excess glucocorticoids exert deleterious effects on the hippocampus and PFC, reducing dendritic spines and altering synapses, changes that are clinically associated with memory deficits and difficulties in emotional regulation (85).

This imbalance helps explain why, in PTSD, some individuals have fragmented or almost absent memories of the trauma but exhibit panic to cue-like stimuli (86).

A fundamental shift came with the work of Alexander and colleagues, who showed that the basal ganglia were not merely a motor module but part of a family of CSTC loops organized in parallel—motor, associative (cognitive), and limbic (emotional) (74). Each loop connects a cortical region to a specific portion of the striatum, then to globus pallidus and substantia nigra, and subsequently to specific thalamic nuclei, before returning to the originating cortical area. The best-known is the motor loop, in which direct and indirect pathways act as “accelerator and brake” of cortical activity, respectively (75).

**Figure 2:**
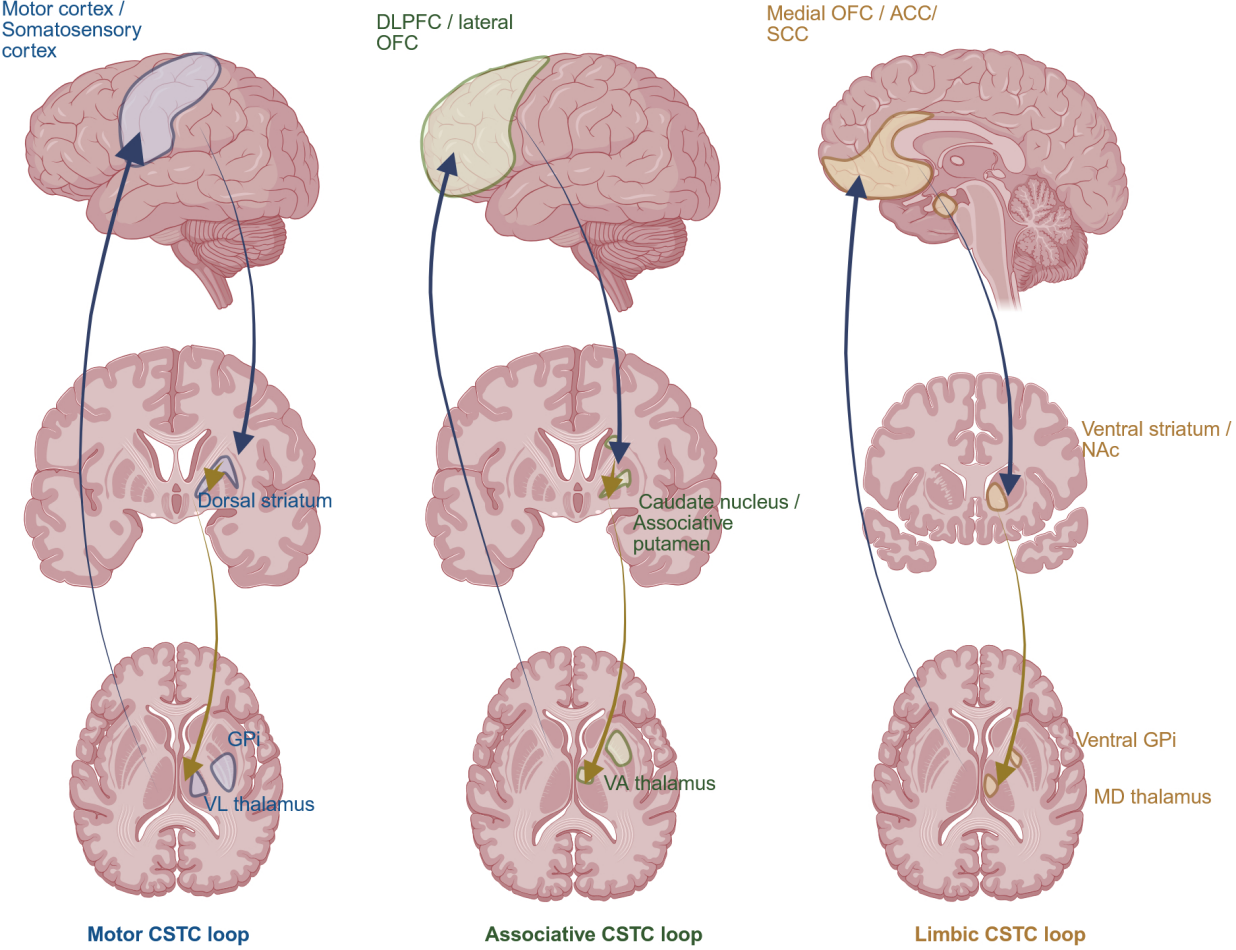
Cortico–striato–thalamo–cortical (CSTC) loops. Schematic representation of the cortico–striato–thalamo–cortical (CSTC) loops. Motor (blue), associative/cognitive (green), and limbic (yellow) circuits are illustrated, reflecting functional segregation within the striatum and thalamus that connects with distinct cortical territories; blue arrows indicate predominantly excitatory projections while yellow arrows indicate predominantly inhibitory pathways. DLPFC, dorsolateral prefrontal cortex; GPi, globus pallidus internus; lateral OFC, lateral orbitofrontal cortex; VA thalamus, ventral anterior nucleus of the thalamus; medial OFC, medial orbitofrontal cortex; ACC, anterior cingulate cortex; SCC, subcallosal cingulate cortex; NAc, nucleus accumbens; MD thalamus, medio-dorsal nucleus of the thalamus.

The associative and limbic CSTC loops are particularly relevant to psychiatry. The associative loop, involving the DLPFC and lateral OFC, the associative striatum, and the ventral anterior thalamus, supports executive functions such as working memory, cognitive flexibility, and plan updating. The limbic loop connects medial OFC and ACC with the ventral striatum (including the NAc), translating the emotional and motivational value of stimuli into tendencies to act (87, 88). Alterations in these circuits have been linked to apathy in Parkinson’s disease (PD), motivational deficits in depression, and dysregulation of reward valuation in addictions (89–92). **Figure 2** schematically illustrates the main CSTC loops involved in motor, associative, and limbic functions.

Although these circuits were initially described independently, they share key nodes and neurotransmitters. In a highly simplified way, the limbic loop “switches on” the desire to act, the associative loop organizes the plan, and the motor loop executes the movement. Within this network, the STN is often regarded as an integrative node or “clock” of the basal ganglia.

Physiological studies show rhythmic firing patterns in the STN that synchronize basal ganglia and cortical activity (93, 94). More recent work in humans suggests that it adjusts the threshold for initiating or stopping decisions and movements, integrating limbic, cognitive, and motor signals before action (95). Since the 1980s, the NAc has been described as an interface between desire and action (76), integrating emotional value, cognitive context, and response options to facilitate action selection, rather than acting as a simple “pleasure center” (96, 97).

#### 3.2.1 Neurotransmitters

Many emotional and cognitive processes depend on three major monoaminergic neuromodulatory systems: dopamine, serotonin, and noradrenaline. Unlike “fast” neurotransmitters such as glutamate or GABA, which act at highly localized synapses, these amines are synthesized in small brainstem and midbrain nuclei —VTA, the substantia nigra pars compacta (SNc), raphe nuclei, and locus coeruleus (LC) —and project diffusely to the limbic system and cortex. This broad projection pattern supports the modulation of motivational, affective,and arousal states, rather than discrete information transmission.

Dopamine is crucial for motivation, reward-based learning, decision-making, working memory, and motor regulation. From the VTA, dopaminergic neurons form the mesolimbic pathway, projecting to the NAc and other limbic structures, which are considered the core of the reward system. Coordinated activity in the VTA–NAc axis guides reward-seeking behavior and contributes to vulnerability to addiction, as virtually all addictive drugs increase dopamine levels in the NAc and redirect motivation toward the drug (98–100). The mesocortical pathway, also originating in the VTA and terminating in the PFC, participates in executive functions; disruptions have been linked to negative and cognitive symptoms of schizophrenia and attention difficulties across various disorders. The nigrostriatal pathway (SNc to dorsal striatum) is essential for CSTC motor loops, while the tuberoinfundibular pathway inhibits prolactin secretion.

Within dopaminergic circuits, the medial forebrain bundle (MFB) acts as a major fiber highway connecting the VTA, NAc, and PFC. In animal models, self-stimulation of the MFB is preferred over basic behaviors such as feeding or resting, illustrating its powerful reinforcing properties (101, 102).

In humans, DBS of the MFB for TRD has been associated with sustained antidepressant effects, consistent with Panksepp’s conception of the MFB as the core of a “seeking system”: a circuit involved in exploration, curiosity, and reward anticipation, whose hypoactivity may manifest as anhedonia and apathy (103, 104).

Serotonin (5-HT) is synthesized primarily in the raphe nuclei of the brainstem, which send descending fibers to the spinal cord (modulating nociception and motor control) and ascending projections that influence affect, sleep–wake cycle, thermoregulation, appetite, and pain perception. Recent studies reveal substantial heterogeneity among serotonergic neurons, with sometimes opposing effects: depending on connectivity and receptor profiles, serotonin can promote approach, avoidance, or anxiety regulation (105, 106).

Noradrenaline arises mainly from the locus coeruleus, a small pontine nucleus with widespread cortical and subcortical projections. The noradrenergic system is fundamental to the stress response and the brain’s “alarm” system: it increases arousal, focuses attention on salient stimuli, and coordinates responses via the sympathetic nervous system and the hypothalamic– pituitary–adrenal axis. LC activation increases noradrenaline release in the cortex and amygdala, enhancing vigilance, encoding of emotional memories, and motor preparedness; reduced activity lowers alertness and environmental reactivity (107).

#### 3.2.2 Connectomics and psychiatric disorders

Connectomics posits that understanding mental health and psychiatric disorders requires mapping structural and functional connections linking different brain areas—the *connectome—* using advanced MRI techniques such as diffusion tensor imaging (DTI) and functional MRI (fMRI). DTI measures how water molecules diffuse within a given tissue (108). In white matter, myelin sheaths constrain water to move preferentially along axonal tracts (anisotropic diffusion), allowing the inference of fiber orientation and the reconstruction of 3D images known as tractography (109), which measures structural connectivity.

Functional connectivity can be inferred with fMRI, which exploits the blood oxygen level– dependent (BOLD) signal to identify brain areas that co-activate during tasks or in pathological states (110). Resting-state fMRI (rs-fMRI) analyzes spontaneous brain activity in the absence of explicit tasks, delineating functional networks such as the default mode network (DMN), active during introspection, self-reflection, and autobiographical memory (111). rs-fMRI has been widely used in psychiatry to describe altered connectivity patterns in several disorders relative to healthy controls (112).

Once maps of structural and functional connectivity are obtained (113), the brain can be represented as connectivity matrices and analyzed with graph theory to build computational models whose dynamics are tuned to reproduce observed neuroimaging patterns (114).

Abnormalities in these measures have been reported in disorders such as schizophrenia, involving both structural and functional connectivity alterations (115, 116).

Despite high expectations (potential role in biomarkers, diagnostic or prognostic tools), the impact of connectomics on routine clinical practice remains limited. The main reasons are the complexity and variability of the connectome-connections vary across individuals and change over time; there is no single “healthy connectome”-, and technical and conceptual limitations: spatial and temporal resolution of fMRI and DTI, heterogeneity in psychiatric diagnoses with symptom overlap, and difficulties of modeling psychiatric disorders in animals. Finally, identifying an abnormality does not imply causality; observed alterations may represent consequences, epiphenomena, or compensatory mechanisms (117). Moreover, diffusion MRI tractography is constrained by well-known pitfalls: reconstructed streamlines are model- dependent estimates rather than direct measurements of axons, and results can vary with acquisition, preprocessing, model choice, and tracking parameters. Crossing fibers, partial volume effects, and distance-related biases may yield false positives/negatives, limiting individual-level inference. Accordingly, connectomic associations with clinical response should be interpreted as predictive markers rather than definitive mechanistic proof, and ideally replicated across centers.

## 4 Stereotaxy and surgical techniques

Contemporary psychosurgical interventions can be broadly categorized into two major groups: lesioning (ablative) techniques and stimulation strategies. Both share a fundamental principle: to intervene as little as necessary to restore a dysfunctional circuit. The choice between them depends on the specific indication, center experience, and patient preferences, weighing the irreversibility and relative simplicity of lesioning against the reversibility, adjustability, and costs associated with stimulation.

Most procedures are based on stereotactic surgery, which treats the brain as a three- dimensional map in which all structures can be described using a Cartesian coordinate system and accessed with millimetric precision. Stereotactic neurosurgery relies on frame-based systems anchored to the skull and on the fusion of neuroimaging modalities (high-resolution MRI and intraoperative stereotactic CT), which, through dedicated software, allow the calculation of coordinates and safe trajectories to each target (118). In recent years, stereotactic frames have begun to be replaced by robotic devices. Robot-assisted surgery has shown, in several meta-analyses, that it is not inferior to frame-based approaches in terms of accuracy (119, 120). Target definition combines indirect methods, based on stereotactic atlases for structures that are poorly visualized on MRI (121), and direct methods based on each patient’s anatomy, thereby reducing errors due to interindividual variability.

### 4.1 Lesions

Lesioning techniques aim to modulate abnormal brain circuits through a localized ablation. Although they may appear similar, each modality deposits energy differently and triggers specific biological cascades.

Radiofrequency (RF) thermal ablation uses a stereotactically inserted electrode whose tip delivers a high-frequency alternating current (~500 kHz). This induces ionic agitation and microscopic friction, raising tissue temperature to around 70–85 °C for several seconds, causing coagulative necrosis in a sharply delimited volume that depends on electrode geometry, temperature, duration, and local microanatomy (122). Historically, the most frequently used procedures in psychiatry have been capsulotomy and cingulotomy.

In stereotactic radiosurgery (SRS), exemplified by Gamma Knife surgery (GKS), hundreds of collimated gamma-ray beams are focused onto an isocenter only a few millimeters in diameter, delivering high doses (≈140–180 Gy) to the target, typically the ALIC in radiosurgical capsulotomies. Unlike RF, it does not require intraparenchymal electrodes. Although a purely radionecrotic mechanism was classically assumed, post-mortem studies suggest that destructive tissue damage is insufficient to fully explain clinical improvements, and a radiotherapy-induced neuromodulatory mechanism has been proposed, involving slow changes in glia, microvasculature, and synaptic remodeling that collectively reset circuit tone and may explain the latency of therapeutic effects (123–125).

Laser interstitial thermal therapy (LITT) employs an optical fiber that delivers collimated laser light to the target. The energy is absorbed and converted into heat, which then spreads locally. Real-time MRI-based thermal monitoring estimates the three-dimensional volume of damage as temperature rises, enabling precise titration of the ablation and limiting side effects from injury to neighboring structures (126). As with other techniques, the clinical effect likely combines focal ablation of critical fibers (e.g., in capsulotomy for OCD) with secondary reorganization of fronto–limbic networks (127).

MRI-guided focused ultrasound (MRgFUS) delivers ultrasound through the skull and focuses the energy on a deep target. At the focal point, ultrasound absorption translates into heating (55–60°C), producing thermal necrosis. In addition, mechanical effects may trigger local repair responses (128). MRI thermometry guides energy delivery in real time, in a way analogous to LITT. In psychosurgery, MRgFUS experience is currently limited to capsulotomies for OCD, with improvements comparable to those achieved with classical ablative techniques or DBS (129), again suggesting that the functionally relevant mechanism is circuit modulation rather than tissue destruction alone.

### 4.2 Stimulation

In contrast to lesioning, stimulation techniques modulate circuits without destroying tissue and allow progressive adjustments at the cost of implanted hardware and higher initial and maintenance costs. Although infrequent, complications related to electrodes and generators should also be considered. Three main modalities can be distinguished: deep brain stimulation, vagus nerve stimulation, and cortical stimulation.

DBS involves implanting intracerebral electrodes using stereotactic techniques and connecting them subcutaneously via extension leads to an implantable pulse generator (IPG), usually placed in the subclavicular region or abdomen. Initially conceptualized as a “reversible lesion,” DBS is now better described as a circuit therapy. Electrical pulses trigger action potentials in axons both orthodromically and antidromically, generating new patterns of synaptic activity that overwrite the pathological ones. In addition, repeated stimulation appears to modify neurotransmitter release and glial metabolism, possibly contributing to the maintenance of clinical effects, and electrode implantation itself can induce a transient “microlesion effect”. Connectivity and tractography studies show that clinical improvement correlates more strongly with the stimulated fiber pathways than with the exact anatomical position of the contacts, emphasizing that what is being treated is a network rather than a single nucleus (130).

Modern generators deliver constant-current stimulation, ensuring a stable electric field despite changes in impedance. Newer leads incorporate segmented contacts (directional electrodes) that allow steering of the electric field towards the intended target and away from neighboring structures, thereby reducing the required current and minimizing side effects. Adjustments in amplitude, frequency, and pulse width define the volume of activated tissue, the therapeutic unit of DBS (131).

Lead implantation is performed using frame-based or robot-assisted techniques, often with intraoperative imaging to verify position, with microelectrode recording being barely necessary. After surgery, programming personalizes therapy: contact(s) selection is based on the best balance between clinical improvement and side effects, and parameter adjustment usually starts with high frequency (around 130 Hz), short pulse widths (60–90 μs), and low amplitude, increasing as needed according to clinical response. In movement disorders, changes in stimulation often produce rapid effects, whereas in psychiatry they may take weeks or months, suggesting slower plasticity mechanisms (132, 133).

DBS has a generally favorable safety profile, but risks must be acknowledged. Intracranial hemorrhage occurs in around 1.5% per implanted electrode in large series and meta-analyses (lower if only symptomatic hematomas are considered) (134, 135). System infections occur in roughly 5% of cases, and in about half of these, they require complete hardware removal (136). Side effects from stimulation of neighboring, unintended structures (e.g., hypomania, paresthesias) are usually reversible with parameter adjustments. With newer designs, hardware- related complications such as lead fracture or migration are becoming less frequent (137).

Rechargeable generators have reduced the frequency of replacement surgeries, and devices capable of recording local field potentials (LFPs) open the way to adaptive strategies. Adaptive DBS (aDBS) uses a closed-loop paradigm in which stimulation is modulated in real time based on a disorder-specific biomarker (e.g., beta-band power in the LFPs of STN in PD) (138). Early studies show symptomatic improvement, fewer adverse effects, and energy savings compared with continuous stimulation (139). The main challenge in psychiatry is to identify robust biomarkers, given that pathological signals are more diffuse and heterogeneous (140–142).

In this setting, recent methodological work combining invasive recordings (LFPs) from DBS leads and non-invasive surface electroencephalogram (EEG) data has enabled the study of cortical- subcortical interactions underlying some cognitive and emotional processing in humans (143).

Vagus nerve stimulation (VNS) exemplifies serendipity in clinical practice. Developed originally for drug-resistant epilepsy in patients not suitable for resective surgery, many recipients later reported mood improvements that exceeded seizure control, leading to specific trials in TRD, and FDA approval for this indication in 2005 (144). Technically, VNS systems are relatively simple: a helical electrode is anchored to the cervical vagus nerve and connected to a subclavicular IPG. Intermittent pulses ascend to the nucleus tractus solitarius (NTS) in the brainstem, a hub with connections to limbic structures involved in seizure generation and mood regulation. Although the antidepressant mechanism of VNS is not fully understood, it has been shown to modulate fronto–limbic networks and monoaminergic circuits (145, 146).

Repetitive transcranial magnetic stimulation (rTMS) lies at the least invasive end of the stimulation spectrum, as it does not require surgery or permanent implants. It is very safe and associated with minimal side effects; however, its therapeutic effects are more modest than those of invasive interventions and require repeated sessions to maintain clinical benefits. rTMS uses a coil to generate magnetic fields that induce electric currents in the underlying cortex, modifying neuronal excitability (147). At an initial mechanistic level, repeated stimulation is thought to induce frequency-dependent changes in cortical excitability and synaptic plasticity, which may in turn influence activity across fronto-limbic networks implicated in mood regulation. In psychiatry, high-frequency rTMS over the DLPFC is increasingly used in TRD (148, 149), and is being investigated in other neuropsychiatric indications, including movement disorders, post-stroke rehabilitation, and neuropathic pain (150–152).

In recent years, several new stimulation techniques and protocols have expanded the clinical landscape of TMS. Patterned forms of stimulation, such as theta-burst stimulation (TBS) and particularly intermittent TBS (iTBS) allow shorter treatment sessions while maintaining antidepressant efficacy comparable to conventional rTMS, which has increased their clinical adoption (153). In parallel, accelerated TMS paradigms –delivering multiple sessions per day over a few days rather than once daily over several weeks-- have attracted interest as a strategy to shorten treatment courses and potentially induce faster clinical improvement (154). Taken together, although promising results in severe depression, protocol heterogeneity, limited sham-controlled data for some approaches, and the need for standardization still constrain broad conclusions (155). Overall, these developments reinforce the view of TMS as an evolving group of circuit-based interventions rather than a single fixed technique.

Another emerging modality is low-intensity ultrasound (LIFU), which allows non-invasive neuromodulation of cortical surface or deep brain structures with relatively high spatial precision. Unlike MRgFUS, which is an ablative procedure, LIFU employs acoustic waves to change neural activity without producing tissue lesions. Early experimental and clinical studies suggest potential applications in psychiatric conditions, such as TRD or addictions; although this approach remains largely investigational and requires further validation (156).

Intracranial cortical stimulation, by contrast, involves electrodes positioned over the cortical surface, in epidural or subdural locations, to modulate neural activity. Its main current indication is the treatment of drug-resistant neuropathic pain (motor cortex stimulation) (157), but some groups have implanted epidural electrodes over the DLPFC in patients with refractory depression, particularly in patients who had previously responded to rTMS (158).

Broadly speaking, ablative procedures and DBS have been used primarily in severe, treatment- refractory OCD, TRD, and a limited number of other severe psychiatric conditions. By contrast, TMS and VNS are particularly used in depressive disorders. Regulatory status nonetheless varies across techniques and indications, and several interventions discussed in this review remain investigational in psychiatry. **Table 1** summarizes the main procedures discussed, together with the corresponding indications and their current regulatory status, where applicable.

**Table 1:** Neurosurgical and neuromodulation procedures: principal psychiatric indications and current regulatory status.

Procedure	Main psychiatric indications	Regulatory status	Notes
Deep brain stimulation (DBS)	OCD, TRD, TS, exploratory use in AN, addiction, aggression, schizophrenia, PTSD	FDA HDE for severe treatment-refractory OCD; investigational for other psychiatric indications	Multiple targets including ALIC/VC-VS, BNST, SCC, NAc, STN.
Ablative procedures (capsulotomy, cingulotomy)	OCD, TRD	Available in specialized centers; not broadly FDA-approved for psychiatric indications	Includes RF, SRS, MRgFUS, and LITT
Vagus nerve stimulation (VNS)	TRD	FDA-approved for chronic or recurrent depression after failure of multiple treatments	Adjunctive therapy; long-term observational studies suggest progressive improvement.
Repetitive transcranial magnetic stimulation (rTMS)	TRD, OCD, AN	FDA-cleared for TRD and OCD	Includes protocols such as theta-burst and accelerated stimulation.
Ultrasound neuromodulation (LIFU)	Experimental neuropsychiatric applications	Investigational	Emerging non-invasive techniques capable of modulating deep brain circuits.

Summary of the main surgical and neuromodulatory techniques addressed in the manuscript. For each intervention, the most relevant indications and the current regulatory status are indicated to provide an overview of the clinical context in which these therapies are currently used or investigated. ALIC, anterior limb of the internal capsule; AN, anorexia nervosa; BNST, bed nucleus of the stria terminalis; DBS, deep brain stimulation; FDA, Food and Drug Administration; HDE, Humanitarian Device Exemption; LIFU, low-intensity focused ultrasound; LITT, laser interstitial thermal therapy; MDD, major depressive disorder; MFB, medial forebrain bundle; MRgFUS, magnetic resonance-guided focused ultrasound; NAc, nucleus accumbens; OCD, obsessive–compulsive disorder; PTSD, post- traumatic stress disorder; RF, radiofrequency; rTMS, repetitive transcranial magnetic stimulation; SCC, subcallosal cingulate cortex; SRS, stereotactic radiosurgery; STN, subthalamic nucleus; TPS, transcranial pulse stimulation; TRD, treatment-resistant depression; VC/VS, ventral capsule/ventral striatum; VNS, vagus nerve stimulation.

## 5 Evidence by indication

**Figure 3:**
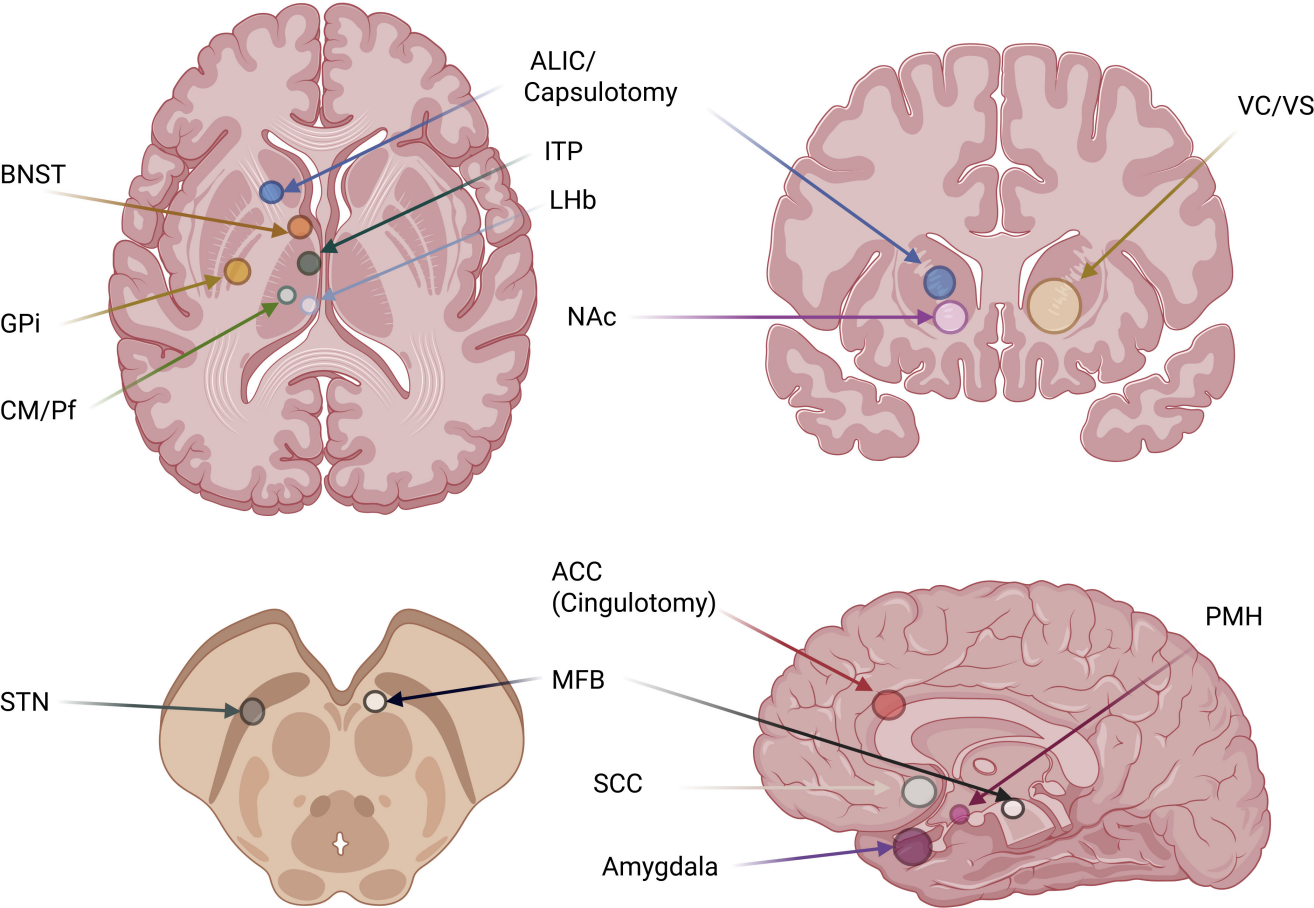
Principal anatomical targets in psychosurgery and neuromodulation. Schematic representation of commonly used targets in DBS and ablative procedures across axial, coronal, mesencephalic, and sagittal planes. ALIC, anterior limb of the internal capsule; VC/VS, ventral capsule/ventral striatum; NAc, nucleus accumbens; BNST, bed nucleus of the stria terminalis; GPi, internal globus pallidus; CM/Pf, centromedian–parafascicular complex of the thalamus; ITP, inferior thalamic peduncle; LHb, lateral habenula; STN, subthalamic nucleus; MFB, medial forebrain bundle; ACC, anterior cingulate cortex; SCC, subcallosal cingulate; PMH, posteromedial hypothalamus.

The clinical translation of the preceding concepts requires examining each disorder in detail, with clear selection criteria, realistic expectations, and a defined follow-up plan. In the sections below, we summarize current evidence and practice in a narrative format aimed at practical use in clinical settings. The main anatomical targets currently employed in psychosurgical and neuromodulatory interventions are schematically depicted in **Figure 3** whereas **Table 2** provides a summary of the main indications, targets, and evidence discussed in this section.

**Table 2:** Indications, targets, and levels of evidence across different disorders in psychosurgery.

Indication	Ablative procedures	Stimulation targets	Evidence summary
Obsessive– compulsive disorder (OCD)	RF/SRS/MRgFUS/LITT capsulotomy; RF/SRS cingulotomy	ALIC, VC/VS, BNST, aSTN, ITP, MFB DBS, connectome-guided DBS	ALIC, VC/VS, BNST, aSTN DBS: most robust evidence; RCTs and long-term cohorts. Long- term efficacy established.
Treatment- resistant depression (TRD)	RF/MRgFUS capsulotomy; RF/SRS cingulotomy	SCC, VC/VS, MFB DBS; VNS	Moderate and heterogeneous evidence: early RCTs yielded mixed results; open-label and long- term follow-up suggest benefit in selected subgroups; strong inter- individual variability.
Eating disorders (AN)	RF capsulotomy	NAc, SCC DBS	Limited evidence: small series and pilot studies in highly selected patients; remains investigational.
Tourette syndrome (TS)	—	CM–Pf, motor and limbic GPi DBS	Moderate evidence: multiple case series and small controlled studies; target superiority remains unresolved.
Treatment- resistant aggression	Historical cingulotomy and hypothalamotomy; NAc ablation (discontinued)	PMH, limbic GPi, NAc DBS	Observational evidence: small heterogeneous series; best prospective data for PMH DBS.
Addiction	Historical cingulotomy and hypothalamotomy; NAc ablation (discontinued)	NAc DBS	Emerging evidence: small cohorts and an initial RCT; promising but experimental.
Schizophrenia	RF capsulotomy	NAc, SCC, habenula, SNr (exploratory)	Sparse and fragmented evidence: small samples, diverse targets; preliminary and experimental.
Post-traumatic stress disorder (PTSD)	—	SCC/uncinate pathway; amygdala DBS	Very preliminary; phase I studies.

Summary of the main psychiatric indications explored in functional neurosurgery, detailing ablative procedures, stimulation techniques, and the current body of evidence for each condition.

RF, radiofrequency; SRS, stereotactic radiosurgery; MRgFUS, MR-guided focused ultrasound; LITT, laser interstitial thermal therapy; DBS, deep brain stimulation; VNS, vagus nerve stimulation; ALIC, anterior limb of the internal capsule; VC/VS, ventral capsule/ventral striatum; BNST, bed nucleus of the stria terminalis; aSTN, anteromedial subthalamic nucleus; ITP, inferior thalamic peduncle; MFB, medial forebrain bundle; SCC, subcallosal cingulate; NAc, nucleus accumbens; CM–Pf, centromedian–parafascicular thalamic complex; GPi, internal globus pallidus; PMH, posteromedial hypothalamus; SNr, substantia nigra pars reticulata; RCT, randomized controlled trial.

### 5.1 Obsessive–compulsive disorder

The typical candidate for invasive neuromodulation or ablative neurosurgical interventions is a patient with severe or extreme OCD of more than five years’ duration who has exhausted first- and second-line treatments. This includes cognitive–behavioral therapy (CBT), several selective serotonin reuptake inhibitors (SSRIs), clomipramine, and augmentation strategies with antipsychotics at adequate doses and durations (159). Severity is usually measured with the Yale–Brown Obsessive Compulsive Scale (Y-BOCS; 10 items, range 0–40)(160). Conventionally, response is defined as ≥35% improvement, and remission as a Y-BOCS score <8. However, the scale is not strictly linear, and its items are not equivalent; a clinically meaningful change in a single domain may substantially improve quality of life while still falling below the 35% threshold. For this reason, complementary assessment of disability (e.g., Clinical Global Impression, Sheehan Disability Scale) is advisable (161, 162) and dichotomous responder/non-responder classifications should be interpreted cautiously.

Pathophysiological studies converge on an imbalance between associative and limbic CSTC loops, with predominance of the direct (excitatory) pathway, leading to orbitofrontal, cingulate, and caudate hyperactivity and reduced dorsomedial prefrontal activity on functional neuroimaging (163, 164). Abnormalities in the anterior cingulate (Papez circuit) may help explain the link between mnemonic processes and repetitive checking or washing-related memories(165). In contrast, dysfunctional amygdala-BNST circuits are implicated in the anxiety component prominent in many patients. More recently, volumetric changes in some structures and global connectivity abnormalities relative to controls have been reported (47, 166, 167), and clinical improvement tends to be associated with a partial normalization of these patterns, irrespective of the therapeutic modality (168). Taken together, these findings support a view of OCD as a disorder of fronto–striato–limbic circuits in which certain white-matter corridors, particularly around the ALIC, act as convergence zones for symptom-relevant pathways.

Within this framework, lesioning techniques—especially capsulotomy—provide a way to interrupt internal capsule fibers related to fronto–limbic hyperconnectivity. Radiofrequency and radiosurgical capsulotomy have shown similar efficacy in several open-label series, with response rates around 50–60% in highly refractory patients (169–171). GKS capsulotomy is now more commonly used, with progressive refinement of lesion and dosing along the ALIC (172), and even a double-blind controlled trial shows significant differences between the active and sham groups (173). However, most studies are single-center and non-randomized, and adverse effects such as apathy, fatigue, and cognitive changes are not negligible. Experience with newer techniques such as MRgFUS and LITT capsulotomy remains limited to small open-label series, but early results are encouraging, with sustained benefits during follow-up (174–177).

Cingulotomy, historically used for its anxiolytic effects and supported by anterior cingulate hyperactivity, has also shown significant improvements in large cohorts, although effect sizes are more modest when modern outcome criteria are applied (178, 179).

Overall, ablative approaches are not inferior in terms of efficacy compared to stimulation techniques (180, 181), with response rates around 50-60% (182), but the evidence base is largely composed of uncontrolled studies, and direct comparative trials remain lacking.

DBS aims to achieve circuit modulation, with the advantage of reversibility and adjustability. The ventral capsule/striatum (VC/VS), which includes the ALIC, was the first target explored, building on prior ablative experience. Initial positive outcomes led to controlled studies and larger case series (183). The largest cohort to date supports the effectiveness and safety of VC/VS-DBS in carefully selected patients, with more than 50% of responders (184). Long-term follow-up suggests that benefits can be sustained over years (185). Over time, the preferred stimulation site within the ALIC has progressively shifted posteriorly as improved anatomical and tractography-based understanding of relevant white matter pathways has redefined targeting strategies. Moreover, the Amsterdam group has illustrated the evolution from “grey matter nuclei” to “white- matter hubs”: starting with stimulation of the NAc, they later observed higher response rates when targeting within the ALIC based on tractography, with better outcomes when stimulating fibers of the MFB as it traverses this tract (186, 187). These findings are consistent with a model in which the critical therapeutic element is the stimulated fiber bundle rather than the nominal nucleus.

The BNST, strongly connected with the amygdala and involved in anxiety and threat responses, has gained empirical support as a surgical target. European groups have reported clinically meaningful improvements across multiple scales; in some series, outcomes have been superior to ALIC stimulation and have persisted at long-term follow-up (188, 189). More recently, a double-blind trial showed significant differences versus sham in the blind phase, with progressive improvement in most patients during the open phase (190). Posterior to the BNST lies the inferior thalamic peduncle (ITP). Small open-label studies have reported response rates around 50%, accompanied by metabolic changes in dysfunctional OCD-related areas (191).

Given the relatively high voltages often required, the volume of activated tissue is large, and overlap with neighboring targets is likely. This reinforces the idea that similar clinical effects can be achieved by delivering energy to different nodes within the same dysfunctional network, particularly where major white-matter bundles converge.

Research on other targets, such as the STN and the MFB, stems from observations in patients operated on for PD with comorbid OCD, in whom electrodes placed medially in the limbic STN were associated with improvement in obsessive symptoms. The STN emerged thus as a potential target given its integrating role within the CSTC loops (93, 94). Stimulation of the limbic portion of the STN is thought to modulate pathological activity within fronto-striatal networks involved in compulsive behaviors, response inhibition, negative cognitive bias, and cognitive inflexibility (192, 193). Studies targeting limbic STN demonstrate superiority over sham in reducing Y-BOCS scores (194), further supporting its role as a key node within the broader network underlying OCD. A randomized trial comparing this target with VC/VS found that STN stimulation was not inferior in terms of Y-BOCS reduction. Greater affective improvement occurred with VC/VS stimulation, whereas greater cognitive flexibility was seen with STN stimulation, suggesting partially distinct mechanistic profiles. In addition, STN stimulation may require longer to achieve its full effect (195), although long-term efficacy has been reported (196). Stimulation of the MFB, a few millimeters medial to the limbic STN and used in depression based on reward- circuit hypotheses, has also been explored. Results are promising, but still based on very small samples (197).

DBS for refractory OCD remains regulated under an FDA Humanitarian Device Exemption (HDE) since 2009. Systematic reviews and meta-analyses support the efficacy of this treatment (198), with global response rates approaching 66%. These findings have led to its inclusion in recent guidelines with level A evidence (199). Long-term follow-up studies suggest that effects are maintained or may even increase over years (200, 201), consistent with slow plastic changes, although a minority of patients shift from responder to non-responder status (185).

Several issues remain unresolved. The optimal candidate profile is not fully known; the only relatively consistent predictors of response are good illness insight and adult-onset OCD (202, 203). Symptom subtype does not clearly predict outcome, although ego-syntonic obsessions (e.g., perfectionism, body image) may be associated with poorer outcomes. Nor is it clear which target is most effective for alleviating a given predominant symptom cluster. Efforts to identify “sweet spots”—anatomical areas where stimulation most strongly correlates with Y- BOCS improvement—have identified two optimal sites across multicenter connectivity studies: one more anterior, in the medioventral ALIC, and another in the BNST–ITP region, whereas stimulation of the NAc itself has been associated with suboptimal results (204).

Psychiatric comorbidities and the risk of cognitive deterioration are also key considerations. Data from the Amsterdam group suggest that DBS remains safe and effective in patients with comorbid bipolar disorder or autism spectrum disorder (205, 206). Moreover, neuropsychological studies have not shown cognitive decline after surgery; on the contrary, some patients—particularly after BNST-DBS—show improved cognitive flexibility (207), which may open a “second window” for psychotherapies and supports framing DBS as an adjunctive component within a multimodal treatment strategy (208).

Surgical interventions produce the most significant average Y-BOCS reductions compared with other modalities, such as psychotherapy or medication (209). Yet, despite their efficacy and favorable safety profile, only a small fraction of eligible patients worldwide is referred for evaluation, in contrast to movement disorders. Suggested explanations include limited awareness among clinicians, stigma related to the historical legacy of psychosurgery, and organisational barriers (210–212).

In recent years, DBS for OCD has shifted from a focus on discrete targets to a connectomic framework in which the critical element is the circuit being modulated. Symptoms and response appear to depend on the stimulation of fronto–striato–thalamo–cortical pathways linking ALIC/VC/VS with prefrontal regions (213, 214). This view reconciles previous findings and proposes a unified network model to guide both electrode placement and programming. In parallel, devices capable of chronically recording local neuronal activity via LFPs open the door to biomarker discovery and aDBS. Although most biomarker work has derived from PD (e.g., beta-band oscillations in the STN) (215), the same neurophysiological frameworks are now being applied to OCD. An illustrative example is the description of a robust ~9 Hz circadian rhythm in the ventral striatum of patients with OCD, which persists in non-responders but is markedly disrupted in responders after stimulation onset (216).

### 5.2 Treatment-resistant major depression

Major depressive disorder (MDD) is now understood as a complex syndrome in which multiple biopsychosocial factors converge on cellular and circuit-level dysfunctions that manifest as emotional and behavioral symptoms (217). Depression is increasingly conceptualized as a disorder of distributed networks, where emotional, cognitive, and autonomic features arise from partially dissociable but interacting circuits with key nodes amenable to modulation (218).

Symptom-based classification alone can be imprecise (219), and it seems plausible that future nosology will incorporate circuit dysfunctions and “biotypes” of depression that better reflect patterns observed in clinical practice (220).

Current treatment encompasses a broad range of psychotherapies and pharmacological agents with diverse mechanisms of action (221, 222). Experience with ketamine and psychedelics is particularly promising (223), although longer-term safety and effectiveness remain to be clarified. Recent work has also explored biological markers that may predict response to pharmacological agents such as ketamine. Neuroimaging and electrophysiological studies suggest that activity and connectivity within fronto-limbic networks –particularly involving the ACC—may be associated with antidepressant response. Although these findings remain preliminary, they illustrate how biomarker-informed approaches may contribute to personalized treatment strategies (224, 225)Based on the results of the STAR*D trial (226), non-invasive neuromodulation such as ECT or rTMS should be considered before invasive approaches in drug-resistant patients (227, 228), and surgical options should be reserved for cases with severe, persistent impairment despite adequate trials of these interventions. Surgical candidates typically present with severe depression on standardized scales, illness duration beyond two years, marked functional impact, and documented resistance to multiple pharmacological regimens and ECT. They may also include patients for whom the latter is not appropriate, not acceptable, or has been declined.

Among ablative techniques, capsulotomy and cingulotomy have the most accumulated experience, although they are performed in a few centers and data derive mainly from selected cohorts (229, 230). Early reports with ultrasound capsulotomy (231) and radiosurgical cingulotomy (232) in small series suggest potential benefit with minimal side effects. Still, numbers are too low to draw firm conclusions, and long-term data are scarce.

In DBS for depression, the best studied targets have been the ventral capsule/striatum (VC/VS, ALIC, NAc), the subgenual cingulate cortex (Cg25, SCC), and the medial forebrain bundle (MFB). Trial designs initially extrapolated from movement disorders —short blinded phases, limited parameter exploration—have probably contributed to early disappointing results and may underestimate the therapeutic potential of carefully optimized stimulation. Increasingly, authors favor a more personalized approach, aligning target selection with the predominant clinical phenotype and allowing extended open-label phases to identify effective configurations, though evidence for such tailoring remains preliminary.

The SCC was the first target tested. It was proposed as a key mood-regulating hub based on hyperactivity in MDD on functional imaging (50). Initial open-label pilot studies, including multicenter cohorts, reported response rates of 36–66% with marked reductions in Hamilton (HAM-D) or Montgomery–Åsberg (MADRS) scores (233–236). These encouraging findings led to the randomized, double-blind BROADEN trial, which did not show significant differences between active and sham stimulation at six months and was subsequently halted because of a low probability of success based on an interim futility analysis (237, 238). Limitations include a short blinded phase and programming restrictions. Subsequent open-label follow-up suggested gradual increases in response rates over 1–3 years, highlighting the importance of trial design and adequate optimization windows. Nonetheless, some patients showed minimal benefit, and considerable overlap existed between the stimulated regions of responders and non-responders. Tractography studies then showed better outcomes (above 80% response rates) when stimulation targeted the convergence zone of several white-matter tracts (forceps minor, cingulum bundle, uncinate fasciculus), suggesting that a “white-matter node” where these fibers intersect may be more relevant than the precise grey-matter location of contacts (239, 240). Building on these observations, the Emory group is currently conducting the TRANSCEND trial, focusing on patients with predominant negative affect and psychomotor slowing, and aiming to replicate, under double-blind conditions, the responder phenotype identified in open-label work (241).

The VC/VS region emerged as a target after beneficial mood effects were observed in patients treated for OCD. VC/VS, including the NAc, is a key node in the reward circuit, with afferents from the VTA and efferents to multiple limbic structures; it was hypothesized that its stimulation could alleviate anhedonia. Initial open-label studies were encouraging (242, 243), but the double-blind RECLAIM trial did not show differences between active and sham stimulation at four months, and was terminated for futility (244). Again, short blinded phases and limited parameter adjustment likely contributed to the negative findings. In contrast, another double- blind trial targeting the same region, but allowing a one-year optimization phase before a crossover comparison, reported significant differences favoring active stimulation, with response rates of 50-60% (245).

The superolateral branch of the MFB (slMFB) connects the VTA with the ventromedial PFC and OFC. Tractography shows that its fibers course through the ALIC (246) and stimulation appears to activate descending glutamatergic projections from the ventromedial PFC to the VTA (247), modulating reward-related tracts in the lower ALIC and offering a plausible strategy against anhedonia. Initial MFB-DBS studies reported rapid and sustained improvements (248, 249), leading to a randomized trial that did not detect acute differences in the first two months, but later showed high response and remission rates with relatively low stimulation intensities and mostly visual or oculomotor side effects (250). Ongoing multicenter trials, such as FORESEE III, have reported promising interim analyses (251), and groups with long-term responder rates(252) are seeking predictive biomarkers of response, including PET metabolic correlates, such as hypometabolism in the caudate and mediodorsal thalamus, in responders with prominent anhedonia. These changes, observed on functional neuroimaging after DBS in responders with prominent anhedonia, may reflect reduced rumination and facilitation of reward-motivated behaviors.

Less-explored targets include the ITP and the lateral habenula (LHb). The ITP connects the mediodorsal and intralaminar thalamic nuclei with the OFC; thalamo–orbitofrontal hypermetabolism has been hypothesized in depression. Small ITP-DBS series suggest outcomes broadly comparable to VC/VS-DBS without major adverse events (253). The LHb links limbic regions with monoaminergic brainstem systems (254) and has been proposed as a modulatory target. Initial case series are promising and report symptom recurrence after incidental stimulation interruption, which argues against a pure placebo- mediated effect (255).

More recently, paradigms from epilepsy surgery are being translated into circuit-guided therapies for depression. These approaches assume that mood states can be tracked via network activity and modulated by targeted stimulation. Stereoelectroencephalography (SEEG) electrodes implanted in multiple limbic regions are used both to identify activity patterns associated with depressive states and to perform test stimulation while monitoring affective changes. After a mapping period, a closed-loop device (responsive neurostimulation, RNS) is implanted and coupled to electrodes located in regions with the most robust mapping and best stimulation-induced effects (256). The PReSiDio trial (257), currently recruiting, is based on this methodology and proposes an individualized approach. Another protocol combines DBS electrodes in the SCC and VC/VS with frontotemporal SEEG recording electrodes to characterize, at the individual level, limbic–prefrontal dynamics and their modulation by different DBS configurations (258), consolidating a trend towards personalized network-guided interventions rather than fixed “one-size-fits-all” targets.

Vagus nerve stimulation (VNS) is another tool for patients with TRD. Contemporary reviews emphasize that VNS not only engages monoaminergic circuits but also influences synaptic plasticity mechanisms, inflammatory pathways, and large-scale network activity in line with actual views of depression as a disorder of brain networks and immune-neuromodulatory interactions (259). Through afferent projections to the NTS and its connections with limbic and monoaminergic systems, VNS may exert a progressive modulatory effect on mood-related circuits rather than produce an immediate antidepressant response. Early controlled trials were disappointing, partly due to low stimulation intensities and short follow-up periods (260). However, long-term follow-up of those cohorts and additional series, including a multicenter European study, showed a gradual and progressive benefit, with increasing response and remission rates over months and years (261, 262). This delayed but sustained pattern of improvement may partly explain why short-blinded phases have underestimated its therapeutic potential. The largest observational study (n=795, 5- year follow-up) reported cumulative response rates of 68% in the VNS group versus 41% with treatment as usual and remission rates 43% versus 26% (263). Current evidence is being refined by large multicenter trials, such as RECOVER, the largest neuromodulation study in psychiatry to date, which randomized participants to active versus sham VNS for one year, followed by a prolonged open-label phase (264). Although preliminary reports indicate that the primary endpoint was not met, clinically meaningful improvements were observed, including depressive severity reduction, better functioning, and quality of life (265). These findings suggest that VNS may be particularly relevant as a long-term, progressively-acting adjunctive intervention, whose effects may not be fully captured by symptom scales alone.

Taken together, the depression literature illustrates both the promise and the fragility of current evidence. Results vary across targets and trials, and apparent contradictions may reflect differences in trial design, optimization windows, and the degree to which targeting is guided by white-matter convergence zones rather than by coarse anatomical labels. A connectomic, symptom-cluster-based approach may be more fruitful than searching for a single “center of depression, but requires more rigorous, adequately powered, and longer-term studies to be validated.

### 5.3 Eating disorders

Anorexia nervosa (AN) remains the psychiatric disorder with the highest morbidity and mortality, and its pathophysiology is still partially understood. Body image dissatisfaction and caloric restriction arise from a complex interaction among cognition, emotion, and homeostatic regulation: caloric deficit sensitizes dopaminergic circuits and activates hypothalamic pathways that promote food intake, which conflicts with the desire to lose weight. An irrational fear of weight gain can paradoxically lead to further restriction, greater activation of feeding-related circuits, and intensification of the fear of losing control over eating, often resulting in severe malnutrition (266). Neuroimaging studies in AN have shown abnormal activation of regions involved in self-perception and visuospatial processing (insula, parietal cortex, and prefrontal areas) (267, 268).

The obsessive fear of weight gain, intrusive thoughts about body image, and ritualized calorie planning resemble OCD-like symptoms; indeed, between 20–40% of patients with AN also meet diagnostic criteria for OCD (269). This comorbidity suggests partially shared neurobiological mechanisms. Morphological and rs-fMRI studies have reported connectivity abnormalities in networks involving the prefrontal cortex, striatum, thalamus, and insula, with particularly increased connectivity in the limbic CSTC loop (270, 271). Current models emphasize dysfunctional interactions among several circuits (control, reward, salience, and interoception) rather than a single “AN network”, helping to explain the clinical complexity of the disorder and guiding the search for new therapeutic targets (272). Within this framework, key modulatory nodes include the SCC, insula, and NAc; tractography studies have demonstrated altered SCC connectivity in AN compared with healthy controls (273).

Before considering invasive interventions, non-invasive neuromodulation techniques such as rTMS of the left DLPFC and parietal cortex have been explored. Pilot trials and recent meta- analyses suggest that rTMS can produce modest increases in body mass index (BMI) and improvements in some symptom domains, with a favorable safety profile (274, 275).

Lesioning procedures such as anterior capsulotomy have also been used in refractory, life- threatening AN. Open-label series report clinically relevant BMI increases and improvements in comorbidity scales (276), albeit with the usual risks of capsulotomy and without controlled data. From a circuit perspective, these effects plausibly stem from disrupting hyperconnected fronto– striatal–limbic fibers in the ALIC that subserve rigid, compulsive restriction.

DBS has emerged as the main neurosurgical alternative. The most studied targets in AN are the NAc and the SCC. A landmark series of 28 patients undergoing NAc-DBS reported moderate but sustained BMI increases together with significant improvements in anxiety, depression, and social functioning (277). SCC-DBS, first explored by Lozano’s group, yielded similar effects on BMI and comorbid symptoms, accompanied by PET evidence of metabolic changes (278).

Isolated cases of BNST stimulation (279) and sequential capsulotomy after unsuccessful DBS(280) have also been reported. Several systematic reviews and meta-analyses, including over one hundred patients, suggest that DBS for refractory AN is associated with mean BMI increases of roughly 20–25%, along with improvements in quality of life and psychiatric comorbidities, and a low rate of serious adverse events (281). A recent systematic review comparing different targets found broadly similar outcomes, although SCC stimulation may be associated with somewhat greater BMI gains (282). Comparable effects across different but interconnected targets fit a model in which modulating hubs within a shared network is more important than the specific nucleus chosen.

In morbid obesity, experience is much more limited, because most patients can be effectively treated with bariatric surgery. The pathophysiology combines hyper-responsivity to reward cues, automated eating habits, and, in some subgroups, hypothalamic dysregulation (283).

Accordingly, hypothalamic targets (for hunger/satiety control) and the NAc (to downregulate craving and dysfunctional reward circuits) have been explored. High-frequency stimulation of the lateral hypothalamus has shown modest BMI reductions in small series (281). NAc stimulation, initially undertaken serendipitously in a patient operated on for OCD (284), has yielded only a case report reporting moderate BMI decrease (281). At present, neurosurgical treatment of obesity should be considered purely experimental, ideally within clinical trials and only after failure or contraindication of bariatric surgery (285).

### 5.4 Tourette syndrome

Current pathophysiological models of TS emphasize dopaminergic hyperactivity and resulting synaptic dysfunction within CSTC loops. In TS, there appears to be reduced activity of the indirect pathway in motor and limbic circuits, leading to thalamo–cortical hyperactivity and, consequently, disinhibition of motor and behavioral programs (286, 287).

Functional neurosurgery is reserved for a small subset of patients resistant to conventional management, since many can be reasonably controlled with CBT and antipsychotics, and symptoms often improve or remit after adolescence. Although inclusion criteria vary, surgery is generally considered in young adults with a clear diagnosis of TS, severe and disabling tics as the main source of impairment, and documented resistance to multiple pharmacological and psychotherapeutic treatments (288, 289).

Two main DBS targets account for most of the clinical experience: the centromedian– parafascicular thalamic complex (CM–Pf) and the internal segment of the globus pallidus (GPi).

The CM–Pf complex was initially explored because of its strategic position within CSTC loops(290). It is an intralaminar thalamic nucleus with massive projections to the ventral striatum and frontal cortices, and afferents from the basal ganglia and brainstem, thus integrating motor, cognitive, and emotional information (291, 292). Experimental studies show that CM–Pf efferents strongly modulate striatal activity and action selection, particularly via cholinergic interneurons (293, 294), and thalamic stimulation can decrease dopaminergic transmission(295). Building on this pathophysiological data and on previous use of this target in epilepsy and neuropathic pain surgery (296, 297), Vandewalle and colleagues selected CM–Pf as the first target for TS, reporting marked improvements in tics and aggressive behaviors (298). Subsequent case series, double-blind studies, and connectivity analyses have broadly confirmed and refined these findings, although sample sizes remain modest and methodologies heterogeneous (299–302).

Interest in pallidal stimulation (posteroventral GPi) arose from its efficacy in treating levodopa- induced dyskinesias in advanced PD (303) and from the conceptualization of TS as a hyperkinetic disorder (304, 305). Other groups have targeted the anteromedial (limbic) GPi, based on the view that TS is at least as much a limbic as a motor disorder (306, 307). Comparative studies, however, have not shown major differences between these approaches in terms of Yale Global Tourette Syndrome Scale (YGTSS) score reductions (308). A small randomized exploratory study evaluating simultaneous implantation of pallidal and thalamic electrodes suggested that GPi stimulation might yield somewhat better results, with no clear added benefit from combined stimulation (309).

Still, numbers are too small to support definitive conclusions, but recent meta-analysis yielded significant improvement in tic symptoms (p < 0.001) and psychiatric comorbidities in DBS patients (310).

### 5.5 Treatment-resistant aggression

From a neurosurgical standpoint, candidates for intervention are individuals with treatment- resistant aggression, typically in the context of severe brain damage or neurodevelopmental pathology. These patients present intractable aggressive behavior with recurrent episodes of severe physical harm to self or others, usually abrupt and explosive, despite optimized pharmacological regimens and structured behavioral interventions. The combination of motor, cognitive, and behavioral disturbances often makes management with conventional measures extremely difficult (311).

Historically, surgical treatment of aggression formed part of classical “psychosurgery,” with radiofrequency capsulotomies, amygdalotomies, and cingulotomies performed with variable results and often poorly documented results (21, 312). In the 1970s, Keiji Sano proposed the concept of ergotropic *systems* — structures involved in energy mobilization, such as the posterior hypothalamus, cingulate gyrus, and hippocampus — and suggested stereotactic lesions at different levels of this system to attenuate symptoms (313). The expansion of psychotropic drugs in the 1980s led to a drastic reduction in such procedures, but a small proportion of patients with extreme, refractory aggression has kept the search for neurosurgical options alive, now under much stricter ethical and regulatory standards.

In this context, DBS has re-emerged as an alternative to irreversible lesions, with several targets explored: posteromedial hypothalamus (PMH), amygdala, limbic GPi, and NAc (314–317). Open- label series and observational studies show that, in carefully selected patients, a high proportion experience clinically meaningful reductions on standardized aggression scales and improved quality of life for patients and caregivers, although the evidence remains predominantly from small, uncontrolled series, often with heterogeneous etiologies. The best-studied target is the PMH: prospective studies with long-term follow-up have shown sustained symptom reductions on and, in some patients, improvement of psychiatric comorbidities (314, 318).

In autism spectrum disorder (ASD) with severe intellectual disability, aggression can reach such an extreme that basic care becomes impossible, despite intensive behavioral programs and multiple lines of psychotropic treatment. In this context, in addition to hypothalamic DBS, lesioning procedures such as bilateral anterior capsulotomy and amygdalotomy (by radiofrequency or radiosurgery) have been explored, with series reporting substantial reductions in aggression in most patients (319, 320). A recent review of 18 studies found improvements on validated scales in most cases, but most reports lack controls and long-term follow-up is limited (321).

Ultimately, despite the growing robustness of the literature, neurosurgical treatment of treatment-resistant aggression remains an exceptional option, reserved for extremecases after exhausting well-structured pharmacological and psychosocial therapies.

### 5.6 Addictions

The pathophysiology of addiction is among the best characterized in psychopathology, in part because it can be modelled reliably in animals. Addictions are conceptualized as circuitopathies: addictive behaviors activate the mesolimbic pathway and dopamine release in the NAc, reinforcing stimulus–reward learning (322, 323). With chronic use, neuroadaptations reduce dopaminergic tone, and patients oscillate between use motivated by pleasure (positive reinforcement) and use aimed at relieving withdrawal-related distress (negative reinforcement), a transition framed by Koob and colleagues within the concept of allostasis (324, 325). In addition, the hippocampus and amygdala consolidate memories and environmental cues that trigger craving (326), while mesocortical dysfunction impairs executive control, explaining relapses despite clear awareness of harm (327). From a connectomic perspective, the NAc and adjacent ALIC function as white-matter hubs where reward, salience, and control circuits converge, making them attractive neuromodulatory targets.

The socioeconomic burden of drug abuse and the limitations of detoxification therapies led to the exploration of surgical options in the 1970s, including cingulotomy and hypothalamotomy, with questionable effectiveness and ethical justification (21, 328). These procedures were largely abandoned, with a few exceptions. In the late 1990s, and particularly in China, radiofrequency ablations of the NAc were performed in patients with heroin addiction, with reported abstinence rates of 50–60%, but this practice was later discontinued because of safety concerns and ethical criticism (329).

In the modern era, attention has shifted to NAc-DBS. Multiple case series and an initial randomized trial have reported reductions in craving and substance use across substances, including alcohol, opioids, cocaine, and nicotine (330–332). Less invasive approaches, such as LIFU targeting the NAc, are also being explored: early work suggests that the technique is safe and feasible. In two participants receiving higher doses, it reduced craving compared with sham stimulation (333). At present, all these interventions should still be considered experimental and confined to rigorously monitored clinical trials.

### 5.7 Schizophrenia

Schizophrenia is widely regarded as a neurodevelopmental disorder in which genetic and environmental factors converge to disrupt the maturation of key brain circuits. It can be usefully conceptualized as an imbalance in excitatory–inhibitory tone and abnormal signaling within networks involved in cognition and threat detection, giving rise to positive and negative symptoms and explaining the characteristic evolution from the prodromal phase to chronic stages (334, 335).

The link between schizophrenia and neurotransmitter dysregulation has been documented since the late twentieth century. The dopaminergic hypothesis remains central, supported by the mechanism of action of most antipsychotics and by functional neuroimaging. A glutamatergic hypothesis has been incorporated, with several models proposing hypofunction of glutamatergic NMDA receptors, particularly in specific inhibitory interneurons (336). Supporting evidence includes the psychotomimetic profile of ketamine and phencyclidine, NMDA receptor antagonists that can induce positive, negative, and cognitive symptoms resembling those of schizophrenia (337) and the finding of elevated glutamate synthesis in the anterior cingulate of antipsychotic non-responders versus responders and healthy controls (338). These data collectively support a network-based view in which neurotransmitter abnormalities destabilize large-scale circuits rather than a single region.

Structural brain abnormalities are well documented, but diffuse. Meta-analyses report global reductions in brain volume and decreased volumes in the superior temporal gyrus, fusiform gyrus, hippocampus, and multiple frontal areas (339). Aberrant connectivity and failure of DMN deactivation are also key concepts: the DMN, normally active at rest and deactivated during external tasks, appears insufficiently “switched off” in schizophrenia (340, 341). Clinically, this may favor persistent self-referential and ruminative activity, leading to intrusive thoughts and verbal hallucinations.

This renewed focus on dysfunctional circuits has stimulated interest in neurosurgical treatments, given the substantial proportion of patients refractory to pharmacotherapy and non-invasive neuromodulation such as ECT or rTMS. Overall, published results remain scarce and involve multiple different targets (342).

Historically, ablative procedures such as anterior capsulotomy and subcaudate tractotomy were used (343). A large capsulotomy series reported approximately 75% improvement in Positive and Negative Syndrome Scale (PANSS) scores in 116 patients, with the expected side-effect profile for this procedure (344). These figures, however, come from an uncontrolled historical cohort and should therefore be interpreted with caution.

Several DBS targets have been explored since. The NAc has long been implicated in aberrant salience—the excessive significance attributed to neutral stimuli that favors delusion formation(345). Some groups have therefore targeted the NAc, reporting greater impact on positive symptoms (346, 347), although numbers remain small and long-term outcomes are uncertain. The SCC is another proposed target, given its links with affective states and the DMN; neuromodulation of this region has also shown promising results, with particular efficacy in positive symptoms and clinical worsening during treatment discontinuation (346). From a connectomic perspective, both NAc and SCC lie in fiber convergence zones linking limbic, salience, and control networks, which may explain their impact on psychosis-related symptom clusters.

Stimulation of the habenula has been proposed as another approach: through its connections with the VTA and SNc, it could help normalize aberrant dopaminergic signaling. Open-label series suggest that this may be an effective target (348), but evidence is preliminary. Finally, hyperactivity of the substantia nigra pars reticulata (SNr) has been hypothesized to contribute to hypofrontality by excessively inhibiting thalamocortical circuits (349). To date, a single clinical case has been published in which marked remission of hallucinations was observed with low- voltage stimulation (350).

### 5.8 Post-traumatic stress disorder

PTSD is closely linked to dysfunction within amygdala–prefrontal circuits and abnormal fear- extinction responses. Neuroimaging studies indicate that impaired regulation of the amygdala by medial PFC regions, particularly the ventromedial PFC and ACC, contributes to the persistence of pathological fear responses. These findings highlight fear-extinction networks as potential targets to modulate dysfunctional emotional circuits (351, 352). Murine PTSD models demonstrate dysregulation of the amygdala–hippocampus–medial PFC circuit, which impairs fear extinction and maintains a hyperresponsive threat system (353, 354). First-line treatments remain trauma-focused psychotherapies and psychotropic medications (355, 356), while techniques such as rTMS show modest and heterogeneous benefits (357).

In refractory cases, DBS has emerged as an experimental strategy to rebalance prefrontal control and amygdala reactivity. Phase I studies and small case series targeting the SCC, the uncinate fasciculus (linking the amygdala with the ventromedial PFC), or the amygdala itself have reported clinical improvements, but sample sizes are very limited and no controlled trials are available (358, 359). Both DBS and modern closed-loop systems (360) represent promising options for treatment-resistant PTSD, but their use remains restricted to research settings.

## 6 Ethical considerations

Contemporary psychosurgery is only ethically acceptable if it strictly respects the four principles of bioethics—autonomy, beneficence, non-maleficence, and justice—in highly vulnerable psychiatric patients (361), whose capacity to consent may be impaired and should be assessed with specific tools (362). Limited worldwide experience and the scarcity of class I–II evidence for most procedures complicate the provision of fair, clear, and balanced information to patients and families (16). In this context, an additional ethical concern is the risk of therapeutic misconception, whereby patients may interpret participation in clinical trials as individualized care, particularly when neurosurgical options are perceived as a last therapeutic intervention. This highlights the need for informed consent procedures that clearly convey the experimental nature of some interventions, the uncertainty of outcomes, and a realistic balance of potential benefits and risks.

Historical controversies and abuses in prisoners and minors led the National Commission for the Protection of Human Subjects, in 1977, to recommend significant restrictions on psychosurgery (363, 364). In current practice, ethical tensions persist at multiple levels: social (pressures to control aggression or addictions), medical (research enthusiasm), industrial (device-related economic interests), and personal (concerns about identity and integrity). In addition, DBS is among the most expensive medical therapies and requires rigorous cost–effectiveness and equity analyses when proposed for treatment-resistant psychiatric disorders (365).

## 7 Discussion and future directions

Psychosurgery has evolved from crude, non-selective lesions to a set of highly targeted interventions aimed at modulating dysfunctional circuits. Across disorders, a common theme emerges: neither symptoms nor therapeutic effects can be reduced to single structures. Instead, they arise from the dynamics of fronto–striato–thalamo–cortical and limbic networks in which key hubs—such as the ALIC, SCC, NAc, and BNST —are particularly amenable to intervention.

Lesioning techniques and neuromodulation should therefore be understood as complementary ways of modifying aberrant networks, rather than mutually exclusive alternatives.

From a clinical standpoint, OCD and TRD are the indications with the most robust evidence, including controlled trials and long-term follow-up. In OCD, both anterior capsulotomy and DBS of VC/VS, BNST, and related fiber pathways show response rates that are clinically meaningful in a population otherwise at the ceiling of conventional treatments. In TRD, the picture is more heterogeneous: early randomized trials of SCC and VC/VS DBS were negative at short-term endpoints, but open-label follow-up and refined targeting based on tractography suggest that a substantial subgroup can achieve sustained benefits when given sufficient time for parameter optimization. In AN, refractory aggression, TS, addictions, schizophrenia, and PTSD, data remain limited and primarily derive from small, often single-center series. Yet, convergent findings indicate that circuit-based interventions can be life-changing for carefully selected patients.

A key lesson across indications is that time and plasticity matter. In movement disorders, changes after DBS often occur within seconds to days, which historically shaped trial designs with short blinded phases. In psychiatric disorders, improvements frequently emerge over weeks or months, likely reflecting slower synaptic plasticity, network reorganization, and psychological adaptation. Trial designs that ignore this temporal dimension risk underestimating efficacy. Future studies should therefore prioritize longer optimization phases, allow flexible programming, and consider delayed primary endpoints.

Another emerging theme is the shift from target nuclei to target networks. Traditional approaches defined targets in stereotactic coordinates based on group atlases. More recent work relies on tractography and functional connectivity to identify patient-specific white matter bundles whose modulation best predicts response. The concept of “white matter sweet spots”—for example, within SCC or ALIC, where multiple tracts converge—illustrates this transition from gray-matter centric models to a connectomic view.

At the same time, while current regulatory frameworks, clinical trial designs, and reimbursement systems remain largely organized around categorical diagnostic entities, emerging neurobiological evidence suggests that several symptom domains may share common circuit substrates across diagnostic boundaries. Thus, a symptom and circuit-informed framework may represent an important future direction for precision psychiatry, even if major regulatory, methodological, and organizational challenges still limit its implementation in clinical practice. In practical terms, this implies that future psychosurgery will increasingly rely on individualized planning that integrates structural MRI, tractography, and, where feasible, rs- fMRI. Multicenter consortia and open datasets will be critical to validate these connectivity- based predictors.

The development of devices capable of chronic sensing is likely to catalyze a second transformation: from open-loop to adaptive, biomarker-guided neuromodulation. LFP recordings in movement disorders have already identified electrophysiological signatures that can be used to trigger or titrate stimulation in real time. Early work in OCD and depression suggests that circadian patterns and specific frequency bands in ventral striatum or other limbic hubs may differentiate responders from non-responders and track clinical states. In the coming years, research will be needed to define which signals are reliable, specific, and practical in psychiatric populations, and to translate them into closed-loop algorithms that automatically adjust stimulation without compromising safety or autonomy.

At the same time, psychosurgery cannot be viewed in isolation from psychological and social treatments. Across indications, there is growing evidence that neuromodulation may facilitate engagement with psychotherapy or rehabilitation by enhancing cognitive flexibility, motivation, or emotion regulation. In OCD and AN, for instance, improvements in cognitive flexibility and reductions in anxiety may allow patients to re-engage with exposure-based therapies or nutritional rehabilitation after years of failure. Future treatment models should explicitly integrate surgery as one component of a multimodal care plan, with pre- and post-operative psychological interventions designed to capitalize on neurobiological changes.

Ethically and organizationally, psychosurgery occupies a narrow corridor between underuse and misuse. On one side, current referral rates are strikingly low relative to the burden of treatment- resistant illness, resulting in preventable suffering for some patients who might benefit. On the other, the historical record of lobotomies and poorly regulated procedures is a constant reminder of the risks of therapeutic enthusiasm, conflicts of interest, and coercive pressures in vulnerable populations. Robust safeguards—independent multidisciplinary committees, stringent inclusion criteria, standardized assessment of decision-making capacity, and long-term follow-up—are essential. Equally important is transparent communication with patients and families about uncertainties, realistic expectations, and the experimental nature of many interventions. Economic and equity considerations add another layer of complexity. DBS and related technologies are among the most expensive medical interventions. As indications expand into psychiatry, healthcare systems will have to balance individual benefit against costs, and ensure that access does not depend solely on geography or socioeconomic status.

Prospective health economic evaluations, including cost–utility analyses and modeling of long- term outcomes, should accompany clinical studies.

This review has limitations that mirror those of the underlying literature. Much of the evidence consists of small, often heterogeneous series with limited control conditions and variable follow- up. Publication bias, differences in programming strategies, and evolving diagnostic criteria further complicate comparisons across studies. We did not perform a formal systematic review or meta-analysis, and some emerging techniques and targets may not yet be fully captured.

Nonetheless, by integrating historical, anatomical, technological, and clinical perspectives, we hope to provide a coherent framework for understanding contemporary psychosurgery as a network-based, gradual, and multimodal intervention for well-selected subgroups of treatment- resistant patients.

Looking ahead, the field will likely be shaped by three converging trends. First, patient selection may be progressively refined through the integration of clinical phenotypes with emerging biomarkers and connectomic signatures. Second, neurosurgical interventions will likely become more deeply integrated within multimodal treatment pathways, including both ablative procedures (such as GKS, MRgFUS, and LITT) and invasive neuromodulation techniques (such as DBS and VNS) in combination with psychotherapy, pharmacology, and rehabilitation. At the same time, non-invasive neuromodulation techniques will likely continue to evolve, expanding the therapeutic armamentarium. TMS, in particular, including newer stimulation protocols, as well as emerging ultrasound-based approaches such as LIFU and transcranial pulse stimulation (TPS), may open new possibilities for selected neuropsychiatric conditions. Third, further progress will depend on the consolidation of shared ethical, regulatory, and economic frameworks, together with greater consensus across centers on indications, target definitions, surgical approaches, programming strategies, and outcome measures. Equally important will be a deeper mechanistic understanding of how different techniques influence brain networks, both to optimize treatment delivery and to ensure that these interventions are offered as carefully regulated, evidence-informed options for patients who have exhausted other therapeutic alternatives. In this context, computational modelling integrating connectomic data with models of oscillatory dynamics and neuroplasticity are emerging as promising tools to simulate the longitudinal effects of neuromodulation such as DBS, including stimulation onset, withdrawal and long-term adaptation. Such frameworks may help to account for interindividual variability in treatment response and to inform more personalized, circuit-based neuromodulation strategies (366).
